# Supplementary material for: Effect of a Topical Collagen Tripeptide on Antiaging and Inhibition of Glycation of the Skin: A Pilot Study
Source: Int J Mol Sci. 2022 Jan 20;23(3):1101. doi: 10.3390/ijms23031101 (PMC8835374; doi:10.3390/ijms23031101)

International Journal of
*Molecular Sciences*

MDPI

*Supplementary Materials*

# Effect of a topical collagen tripeptide on antiaging and inhibition of glycation of the skin: A prospective, single-arm clinical trial

**Young In Lee [1,2,†], Sang Gyu Lee [1,†], Inhee Jung [3], Jangmi Suk [3], Mon-Hoe Lee [4], Do-Un Kim [4], Ju Hee Lee [1,2,*]**

[1] Department of Dermatology & Cutaneous Biology Research Institute, Yonsei University College of Medicine, Seoul 03722, Korea; ylee1124@yuhs.ac (Y.I.L.); dltkdrb5658@yuhs.ac (S.G.L.)
[2] Scar Laser and Plastic Surgery Center, Yonsei Cancer Hospital, Seoul 03722, Korea
[3] Global Medical Research Center, Seoul 06526, Korea; ihjung@gmrc.co.kr (I.J.); rose@gmrc.co.kr (J.S.)
[4] Health Food Research and Development, NewTree Co., Ltd., Seoul 05604, Korea; mhlee@inewtree.com (M-H.L.); dkim@inewtree.com (D-U.K.)
* Correspondence: juhee@yuhs.ac; Tel.: +82-2-2228-2080
† These authors made equal contributions.

## 1. Supplementary Table

Supplementary Table S1. Overall clinical studies data outcomes.

| | Improvement (%) | Baseline Mean ± SD | 4 weeks Mean ± SD | *p* value |
|---|---|---|---|---|
| *The periorbital skin roughness* | - 7.394 | 20.774 ± 3.506 | 19.238 ± 3.523 | # $p<0.001$ |
| *The glabella skin roughness* | - 5.557 | 21.395 ± 3.094 | 20.206 ± 2.833 | * $p<0.001$ |
| *The maximum periorbital skin roughnees* | - 4.762 | 191.106 ± 32.583 | 182.005 ± 33.629 | * $p<0.001$ |
| *The maximum glabella skin roughness* | - 5.973 | 169.924 ± 21.574 | 159.775 ± 21.577 | # $p<0.001$ |
| *The depth of periorbital wrinkles* | - 4.918 | 0.061 ± 0.010 | 0.058 ± 0.010 | # $p<0.001$ |
| *Skin density* | 7.210 | 55.655 ± 7.611 | 59.668 ± 7.841 | # $p<0.001$ |
| *Skin collagen strength* | 3.253 | 68.023 ± 5.476 | 70.236 ± 5.140 | # $p<0.001$ |
| *Skin surface elasticity* | 1.961 | 0.816 ± 0.032 | 0.832 ± 0.029 | * $p<0.001$ |
| *Advanced Glycated End products measurement* | - 4.253 | 2.257 ± 0.322 | 2.161 ± 0.289 | # $p<0.001$ |

#*p*: by Wilcoxon signed rank test; **p*: by Paired samples t-test

Supplementary Table S2. Primer sequence for qRT-PCR.

| Gene | Forward primer (5'-3′) | Reverse primer (5'-3′) |
|---|---|---|
| *COLA1A1* | GATTCCCTGGACCTAAAGGTGC | AGCCTCTCCATCTTTGCCAGCA |
| *MMP1* | ATGAAGCAGCCCAGATGTGGAG | TGGTCCACATCTGCTCTTGGCA |
| *MMP3* | CACTCACAGACCTGACTCGGTT | AAGCAGGATCACAGTTGGCTGG |
| *MMP9* | GCCACTACTGTGCCTTTGAGTC | CCCTCAGAGAATCGCCAGTACT |
| *GAPDH* | GTCTCCTCTGACTTCAACAGAG | ACCACCCTGTTGCTGTAGCCAA |

Supplementary Table S3. The total ingredients of Ever Collagen Corrector Collagen Tripeptide Ampoule

| Ingredient name | | |
|---|---|---|
| Water | Helianthus Annuus (Sunflower) Seed Oil | Hydrogenated Polydecene |
| Glycerin | Adansonia Digitata Seed Oil | Hydroxyacetophenone |
| Propanediol | Hyaluronic Acid | Hydrogenated Lecithin |
| Isododecane | Hydrolyzed Hyaluronic Acid | Polyglyceryl-10 Oleate |
| Butylene Glycol | Leontopodium Alpinum Callus CultureExtract | C12-20 Alkyl Glucoside |
| Cyclohexasiloxane | Milk Protein Extract | Carbomer |
| Niacinamide | Akebia Quinata Extract | Sodium Polyacryloyldimethyl Taurate |
| Diphenyl Dimethicone | Sambucus Nigra Fruit Extract | Biosaccharide Gum-1 |
| Cetyl Ethylhexanoate | C14-22 Alcohols | Tromethamine |
| Silica | Glycereth-26 | Maltodextrin |
| Triethylhexanoin | 1,2-Hexanediol | Saccharide Hydrolysate |
| Hydrolyzed Fish Skin Extract | Glyceryl Stearate | Adenosine |
| Tocopherol | Sorbitan Stearate | Ethylhexylglycerin |
| Betaine | Dimethicone/Vinyl Dimethicone Crosspolymer | Hydroxyethylcellulose |
| Disodium EDTA | Ammonium Polyacryloyldimethyl Taurate | Rosa Damascena Flower Water |
| Trideceth-10 | Soluble Proteoglycan | Caprylyl Glycol |
| Sodium Hyaluronate | Glucose | Fragrance |

## 2. Supplementary Figure

Supplementary Figure S1. Cell cytotoxicity of EverCTP™ depending on the concentration in the HDF cell. There was no cell cytotoxicity effect on 10, 250, 500 and 1000 μg/ml EverCTP™ treatment.

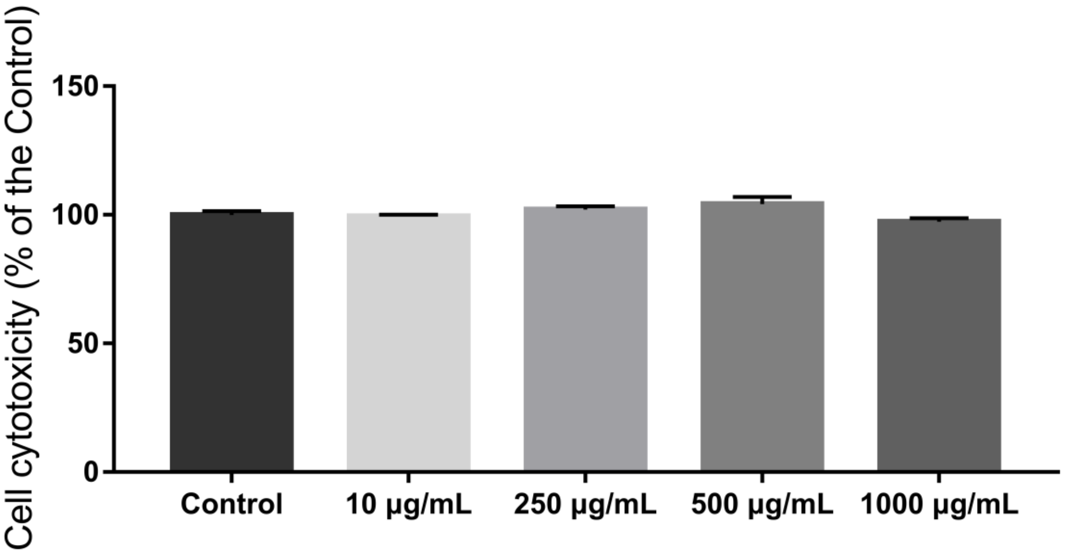

Supplement: Supplementary file 1 [file ijms-23-01101-s001.zip › Supplementary Figure_final.pdf]
