# Supplementary figures and images for: Effect of a Topical Collagen Tripeptide on Antiaging and Inhibition of Glycation of the Skin: A Pilot Study
Source: Int J Mol Sci. 2022 Jan 20;23(3):1101. doi: 10.3390/ijms23031101 (PMC8835374; doi:10.3390/ijms23031101)

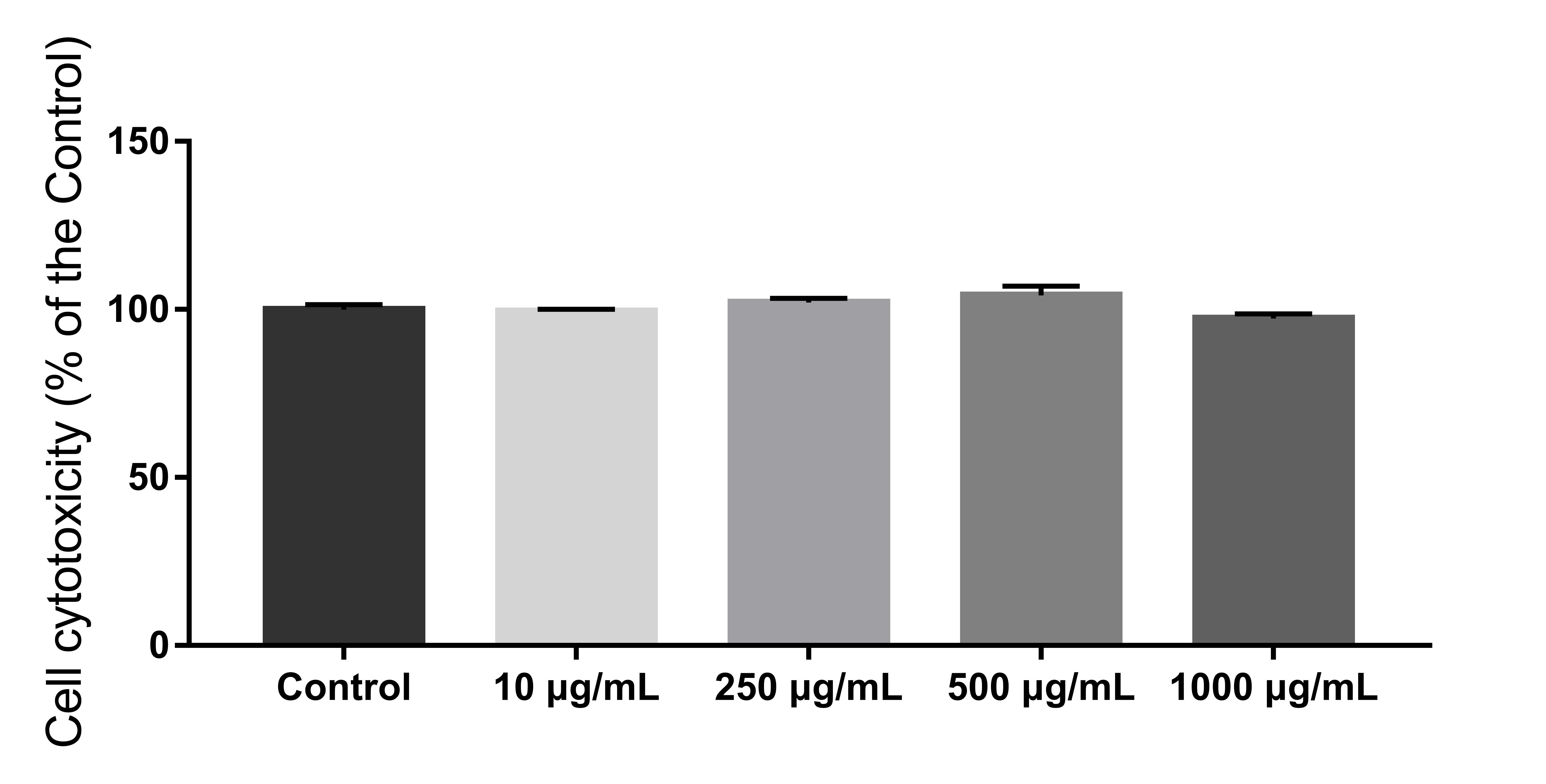

Supplement: Supplementary file 1 [file ijms-23-01101-s001.zip › Supply figure. S1.tif]
